# Laboratory testing improves diagnosis and treatment outcomes in primary health care facilities

**DOI:** 10.4102/ajlm.v1i1.8

**Published:** 2012-10-30

**Authors:** Jane Y. Carter, Orgenes E. Lema, Magdaline W. Wangai, Charles G. Munafu, Philip H. Rees, Jackson A. Nyamongo

**Affiliations:** 1 African Medical and Research Foundation, Nairobi, Kenya; 2 African Medical and Research Foundation, Kampala, Uganda; 3 Nairobi Hospital, Kenya; 4 Ministry of Health, Kenya

## Abstract

**Objective:**

To determine if use of basic laboratory tests improves diagnosis and treatment outcomes in outpatients attending rural primary health care facilities.

**Setting:**

Six rural health centres in Kenya.

**Design:**

Cross-sectional study to observe change in diagnosis and treatment made by clinical officers after laboratory testing in outpatients attending six rural health centres in Kenya.

**Subject:**

The diagnosis and treatment of 1134 patients attending outpatient services in six rural health centres were compared before and after basic laboratory testing. Essential clinical diagnostic equipment and laboratory tests were established at each health centre. Clinical officers and laboratory technicians received on-site refresher training in good diagnostic practices and laboratory procedures before the study began.

**Results:**

Laboratory tests were ordered on 704 (62.1%) patients. Diagnosis and treatment were changed in 45% of tested patients who returned with laboratory results (21% of all patients attending the clinics). 166 (23.5%) patients did not return to the clinician for a final diagnosis and management decision after laboratory testing. Blood slide examination for malaria parasites, wet preparations, urine microscopy and stool microscopy resulted in most changes to diagnosis. There was no significant change in drug costs after laboratory testing. The greatest changes in numbers of recorded diseases following laboratory testing was for intestinal worms (53%) and malaria (21%).

**Conclusion:**

Effective use of basic laboratory tests at primary health care level significantly improves diagnosis and patient treatment. Use of laboratory testing can be readily incorporated into routine clinical practice. On-site refresher training is an effective means of improving the quality of patient care and communication between clinical and laboratory staff.

## Introduction

Primary health care (PHC) is the first-line contact for medical care, service and advice. In Kenya, PHC is provided by community health workers, and at dispensaries, health centres, and outpatient departments of hospitals. The World Health Organization (WHO) advocates for basic laboratory services to support clinical and public health activities at PHC level^1^ and the government of Kenya has planned for appropriate diagnostic services at all levels of the health care system (Levels 1–6); currently, peripheral laboratories are established mainly down to health centre level (Level 3). Most health centres are situated in rural areas, where the majority of the population lives; however, considerable constraints remain in establishing rural laboratory units and supporting their operation.

Basic laboratory tests may assist in better diagnosis and management of six out of ten of the most common diseases and conditions seen in outpatients presenting to health centres and primary level hospitals.^2,3^ More information is needed on how clinicians working in PHC units utilise laboratory tests for patient management, and which tests are most useful for diagnosing and managing patients in different geographical areas. In an editorial in the *British Medical Journal*, Garner *et al*.^4^ raise the questions: do laboratory tests at this level result in altered clinical decision-making, and does access to laboratory testing actually improve the quality of patient care?

Between 1992 and 1994, the African Medical and Research Foundation (AMREF) Laboratory Programme, in collaboration with the National Public Health Laboratory Services, Ministry of Health, Kenya, conducted an essential laboratory programme feasibility study in seven rural health centres to determine an approach to effective and sustainable diagnostic services at primary health care level. The method for the study has been previously described (3), and the results are presented in another paper.^5^ To address the complete diagnostic cycle, the study addressed both clinicians and laboratory staff. During the feasibility study, a sub-study examined how clinicians used laboratory tests, and compared pre- and post-test diagnosis and treatment to examine the effect of laboratory testing on outpatient management.

## Materials and methods

This was a cross-sectional study observing the work of clinical officers in the outpatient departments of six health centres participating in the essential laboratory programme feasibility study. Each health centre was situated in one rural province in Kenya to reflect variation in climate and accessibility. These were: Isibania (Kuria District, Nyanza Province), Katilu (Turkana District, Rift Valley Province), Kimilili (Bungoma District, Western Province), Mariakani (Kilifi District, Coast Province), Matuu (Machakos District, Eastern Province) and Wanjohi (Nyandarua District, Central Province). A seventh health centre was excluded because of the absence of a full-time clinical officer. The sites and geographical profile of the study health centres are detailed elsewhere.^5^

Before the start of the study, a baseline survey was conducted to determine clinical, laboratory and public health activities and review existing facilities and staffing. Clinical diagnostic equipment and supplies were supplemented to ensure every clinician had access to the following: stethoscope, otoscope, sphygmomanometer, torch, vaginal specula, thermometer, patella hammer, tongue depressors, examination gloves, weighing scales.

Basic laboratory tests were selected according to their potential usefulness in diagnosing and managing the most common diseases and conditions seen in outpatient practice; their operability in resource-limited settings; their rapidity and cost; and the technical skills of clinical and laboratory staff at health centre level. Laboratory tests established at each health centre were: haemoglobin estimation to detect anaemia (haemoglobinometer/haemiglobincyanide method); blood slide for malaria and other blood parasites (Field stain); total white blood cell count (manual, improved Neubauer chamber) to support fever investigation; blood film examination for blood cell morphology and differential white blood cell count (reverse Field stain) mainly to support anaemia and fever investigation; urine microscopy (examination of sediment) to detect urinary tract, sexually transmitted and *S. haematobium* infections; urine chemistry (dipsticks) for urine protein and glucose; stool microscopy (direct, eosin, iodine) to distinguish bacterial and parasitic infection; wet preparations of genital and skin specimens (direct, potassium hydroxide) to detect sexually transmitted infections and fungal skin infections; Gram stain to identify bacterial and fungal infections; Ziehl Neelsen stain (standard and modified) to diagnose tuberculosis and leprosy; dark field illumination to detect spirochaetes in genital ulcers; rapid plasma reagin kit for syphilis screening.

The study physician and laboratory technologist, accompanied by the district clinical officer and district laboratory technologist for each district, visited each health centre for 5 days at the study start to introduce and establish project activities. On-site refresher training addressed improved diagnostic practices through one-to-one training of clinical officers and laboratory technicians during routine outpatient clinics. Flow sheets were developed outlining history taking, physical examination, and selection and interpretation of laboratory tests for the major clinical syndromes seen at primary health care level (fever, pallor, diarrhoea, cough, skin diseases, sexually transmitted infections), based on Standard Treatment Guidelines produced by the Ministry of Health. AMREF designed and produced a poster, ‘Use of essential laboratory tests’,^6^ and clinicians were provided with the following AMREF publications: ‘Communicable Diseases’, ‘Child Health’ and ‘Medicine’.

The study was conducted during 2–4-day support supervisory visits carried out 3–4 times at each site over the two-year period. Subjects were patients attending general outpatient curative clinics with a new condition. Clinical officers were requested to take a brief directed history and perform a targeted physical examination on every patient and to request laboratory tests in every case where results could contribute to diagnosis and/or management. A basic laboratory request form including name, age, sex, patient number, brief clinical notes, tests required, signature of clinician and date was completed for every patient. Patients carried the request form to the laboratory, waited for the results and returned to the clinical officer for a management decision. Laboratory staff collected all specimens except high vaginal swabs, endocervical swabs and some pus swabs, which were collected by the clinical officer. For patients requiring laboratory tests, clinical officers were asked to make a preliminary clinical diagnosis and treatment decision, which were entered in the study record sheet, before referring the patient for laboratory testing. After receiving laboratory results, diagnosis and treatment were amended as required. The study physician sat with each clinical officer and collected data from all consecutive patients attending with a new condition, whether laboratory tests were ordered or not. Due to few clinical officers in the health centres, most children under five years were treated in the maternal and child health clinic and only seriously ill children were referred to the clinical officer. Time taken for laboratory testing and overall patient time in the health facility were not recorded.

The data were analysed by comparing pre- and post-test diagnosis and treatment. Confidence intervals were computed at the 95% confidence level.

## Ethical considerations

Ethical approval for the study was provided by the Ministry of Health, Kenya.

## Results

1134 new patients were recruited into the study. Patients were examined by eight clinical officers in a total of 58 days over two years (ranging from 9 to 11 days at each health centre). The average number of patients seen at each health centre was 189 (range 108 to 339); 849 (75%) patients were more than 12 years of age. Female patients predominated at all health centres except two (Wanjohi 49%, Isibania 46%) (Table 1).

**TABLE 1:** Patient characteristics, profile of laboratory testing and change in patient management.

Characteristics	Sub-characteristics	Patient profile							
		Isibania	Katilu	Kimilili	Mariakani	Matuu	Wanjohi	Total (%)
Age	> 12 years	93	106	176	116	265	93	849	74.9
	5 - 12 years	14	48	31	37	71	20	221	19.5
	< 5 years	1	26	3	29	3	2	64	5.6
Gender	Male	58	87	95	78	142	59	519	45.8
	Female	50	93	115	104	197	56	615	54.2
Number of laboratory tests ordered per patient	Total tests	74	123	120	120	214	53	704	–
	% patients tested	69	68	57	65	63	46	–	62.0 CI 59.2 – 64.9
	1 test	50	74	92	82	175	40	513	72.9 CI 69.4 – 76.1
	2 tests	19	35	18	23	32	9	136	19.3 CI 16.5 – 22.5
	3 tests	5	8	7	11	6	2	39	5.5 CI 4.01 – 7.6
	4 tests	–	4	2	3	1	2	12	1.7 CI 0.9 – 3.0
	5 tests	–	1	1	1	–	–	3	0.4 CI 0.1 – 1.3
	6 tests	–	1	–	–	–	–	1	0.1 CI 0.0 – 0.9
Number of patients with laboratory results	All results	64	109	92	78	137	38	518	73.6
	Incomplete results	3	3	5	4	3	2	20	2.8
	No return	7	11	23	38	74	13	166	23.6
	Diagnosis, management change	37	47	41	35	60	22	242	45.0
**Total**	**108**	**180**	**210**	**182**	**339**	**115**	**1134**	**100.0**

CI, Confidence Interval.

## Case data

Laboratory tests were ordered on 704 (62.1%) patients (range 46.1% – 68.5% at each health centre); 971 tests were ordered (range 1–6 tests per patient, average 1.4); one test was ordered for 513 (72.9%: range 60% - 82%) patients (Table 1). While the study physician was present, 166 (23.5%) tested patients did not return to the clinical officer for a final diagnosis and management decision; 20 (2.8%) patients returned with incomplete laboratory results. Of the 538 tested patients who returned with all or incomplete laboratory results, diagnosis and/or treatment were changed in 242 patients (45% of tested patients; 21% of all patients). Table 1 shows the number of patients at each health centre with all results, complete results, or who did not return to the clinician. Of the 971 tests ordered, there were no results in 250 (25.7%). From the 721 test results, 264 (36.6%) resulted in a change in patient diagnosis, drug treatment or both. Table 2 shows the types of tests ordered and numbers of tests contributing to a change in diagnosis or treatment.

**TABLE 2:** Laboratory tests and their effect on diagnosis and treatment.

Tests	Ordered	Results available		Tests resulting in change in diagnosis/treatment		
*n*	*n*	%	*n*	%	CI
Blood slide	498	400	80.3	157	39.3	34.5	–	44.2
Stool examination	167	105	62.9	46	43.8	34.3	–	53.8
Haemoglobin	91	72	79.1	13	18.1	10.3	–	29.3
Gram stain	59	46	77.9	14	30.4	18.2	–	45.9
Urine microscopy	53	36	67.9	17	47.2	30.7	–	64.3
Wet preparation	32	20	62.5	12	60.0	36.4	–	80.0
Blood-film examination or differential white blood cell count	18	10	55.6	0	0.0	0.0
Urine chemistry	15	14	93.3	1	7.2	0.4	–	35.8
Syphilis screening	15	7	46.7	3	42.9	11.8	–	79.8
Total white blood cell count	11	8	72.7	0	0.0	0.0
Ziehl Neelsen stain	11	2	18.2	0	0.0	0.0
Dark field exam	1	1	100.0	1	100.0	5.5	–	100.0
**Total**	**971**	**721**	**74.3**	**264**	**36.5**	**33.1**	**–**	**40.3**

CI, Confidence Interval.

Tests were grouped according to the purpose of making a diagnosis or defining a clinical syndrome. Table 3 shows the change in diagnosis and treatment made by each group of tests.

**TABLE 3:** Change in diagnosis or treatment after laboratory testing.

Laboratory test groups	Blood slide for malaria (*n* = 400)		Stool microscopy (*n* = 105)		Tests^†^ for STI, (*n* = 73)		Haemoglobin estimation (*n* = 72)		Urine microscopy^‡^ (*n* = 30)	
	*n*	%	*n*	%	*n*	%	*n*	%	*n*	%
Change in diagnosis or treatment	157	39	46	43	32	43	13	18	11	37
Change due to negative results	108	69	26	57	17	53	4	31	7	64
Change due to positive results	49	31	20	43	15	47	9	69	4	36

† Tests for STI were examination of urethral swabs, HVS, ECS, conjunctival swab in neonates, first part urine, darkfield examination of GUD and syphilis screening.

‡ Urine microscopy for UTI and parasites, excluding first-part urine for STI.

### Change in diagnosis

**FIGURE 1:**
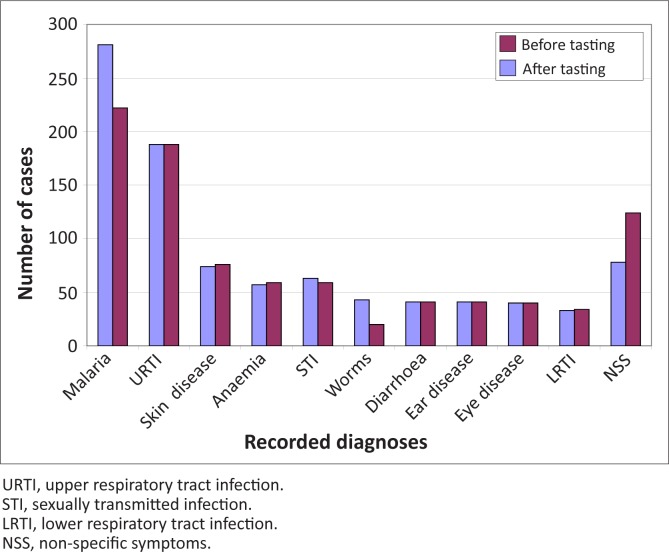
Ten most common diagnoses in all health centres.

Data on diagnosis were grouped according to major diseases and clinical syndromes. The number of diagnoses recorded before and after laboratory testing were compared. Figure 1 shows the 10 most common diagnoses in all health centres, including patients that were not tested in the laboratory.

### Changes in drug use and cost

**FIGURE 2:**
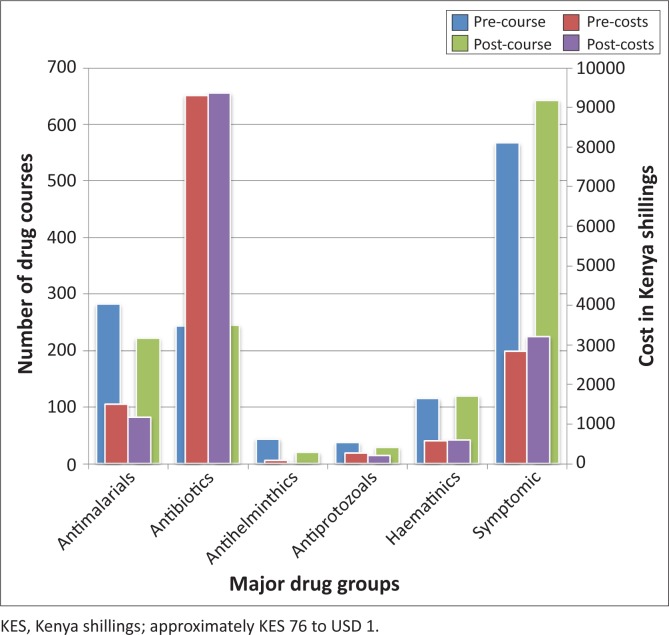
Number of drug courses prescribed and costs before and after laboratory testing.

Drug use indicators were applied to measure the effect of laboratory testing on drug prescription practices.^7^ These were: total number of medicines prescribed; percentage of patients with an antibiotic prescribed; and percentage of patients with an injection prescribed. Table 4 compares drug use indicators before and after laboratory testing. Drugs were grouped into major treatment categories. The number of drug courses prescribed and costs were compared before and after laboratory testing (Figure 2).

**TABLE 4:** Change in drug use indicators after laboratory testing.

Drug use indicator	All patients		Tested patients	
	Before testing	After testing	Before testing	After testing
Average number of medicines prescribed	1.44	1.43 (*p* = 0.377)	1.47 1.45 (*p* = 0.297)
Percentage of patients with antibiotic prescribed	29.1	28.0 (*p* = 0.242)	28.7	27.0 (*p* = 0.294)
Percentage of patients with injection prescribed	6.5	6.1 (*p* = 0.242)	3.8	3.0 (*p* = 0.045)

## Discussion

Many clinics lack essential diagnostic equipment for patient examination. Vaginal specula and swabs were provided in sterile packs in each consultation room to allow immediate vaginal examination. Laboratory investigations took approximately 1 to 3 hours per patient and were usually completed by the end of the morning or afternoon clinic sessions. Clinical officers worked efficiently with all equipment ready to hand and incorporated laboratory testing into their procedures without difficulty. A basic laboratory request form for all tests was appropriate at this level. The clinical impression or provisional diagnosis recorded in the patient’s notes before referring the patient to the laboratory facilitated the final management decision after the laboratory results were received.

Nearly a quarter of tested patients (23.6%) did not return with laboratory results, and 2.8% of patients returned with incomplete results. Although the study supervisors worked in each health centre for 1 to 3 full days at a time, some patients may have returned with results after the supervisors had left. Since the tests are rapidly performed and are intended to provide results before patients leave the facility, investigating causes of delay would be useful. Many health centres were crowded and patients may have become discouraged by the waiting time. Improved organisation of patient flow and adequate numbers of clinical and laboratory staff should increase the numbers of patients who complete investigations. For some tests, such as stool, urine and sputum examination, patients may not have produced specimens; syphilis screening was often processed in batches and patients were asked to return for results on another day. As nearly half the patients referred to the laboratory had a change in diagnosis and or treatment as a result of laboratory testing, it is important to ensure that more patients return to the clinician with completed results.

Clinical officers were advised to interpret laboratory results in the light of patients’ symptoms and signs. In general, the presence of pathology in specimens from symptomatic patients was reported as a positive diagnosis and patients were treated appropriately; however, symptomatic patients with negative test results were also sometimes treated based on the clinicians’ judgement for the following conditions: malaria, intestinal helminthic and protozoal infection, fungal skin infection, pelvic inflammatory disease, or fungal vaginal infection. A change in diagnosis usually resulted in a change of treatment; a few test results altered the diagnosis but not the treatment, e.g. change from *E. histolytica* to *G. lamblia* infection; however, these data may have public health implications. Malaria diagnosis was reduced as a result of laboratory testing; all other diagnoses were increased, due to better assessment of other causes of fever, and the ability of the laboratory to confirm alternative diagnoses. In our study, 222 patients were given the diagnosis of malaria on the basis of 192 positive blood slides. The over-diagnosis of malaria despite use of diagnostic testing has been demonstrated in other studies^8,9^ and is a major limitation to improved case management and cost savings on treatment.^10^ Using a laboratory confirmed diagnosis of malaria as the reference standard, the sensitivity of clinical malaria diagnosis in this study was 78% and the specificity 39%. Malaria infection lacks specific symptoms and signs, and clinical diagnosis is not improved with better history taking.^11^ There was no difference in blood-slide positivity rate in the presence (48.6%) or absence (47.8%) of another condition causing fever (*p* = 0.872), based on physical examination or basic laboratory testing. This finding has been reported elsewhere^12^ but requires further study.

Symptomatic patients with negative endocervical swabs were treated for pelvic inflammatory disease, as endocervical smear microscopy is not a sensitive predictor of disease.^13^ Based on the sum of results from a group of tests, the sensitivity of clinical diagnosis of sexually transmitted infections was 65%. The test that made the most difference to diagnosis was examination of wet preparations of high vaginal swabs (53% change in diagnosis); examination of sediment of first part of morning urine to rule out urethritis changed the diagnosis in a single symptomatic male patient. These findings have major implications for continued use of syndromic approaches as practised by some disease control programmes. Testing to determine specific diagnoses is recommended to reduce cost of treatment and improve compliance.^13^ Syndromic management would be made more effective if tailored to the skills of clinical staff and available laboratory investigations at different health facility levels.

The ability of a laboratory test to change diagnosis is an important measure of usefulness, but is not the only factor. In general, tests were used to confirm a suspected diagnosis, but tests performed to rule out conditions are also of value. Based on the number of patients for whom laboratory tests altered diagnosis and the clinical and public health importance of the diseases confirmed, a selection of core tests can be drawn up for an outpatient health service. In this study, total and differential white cell count and thin blood film examination made little difference to diagnosis or patient management, although they were helpful in providing a more complete clinical picture. Potassium hydroxide preparation and dark field examination, although useful, address few cases and may be technically more demanding. Ziehl Neelsen stain and syphilis screening addressed few cases but are important in diagnosing serious and treatable diseases, and should be retained. In this study setting, patients with suspected tuberculosis or syphilis would have been referred to another facility for laboratory confirmation, so the diagnosis in the health facility records was recorded as ‘changed’.

The change in diagnostic data resulting from improved laboratory use in outpatient services could have a major impact on national health estimates and planning if applied country-wide. For example, in this study malaria diagnosis was reduced by 21%. Change in drug costs before and after laboratory testing was less than 1%, as change in diagnosis generally resulted in the first proposed treatment being changed to another. Selected drug use indicators for primary health care facilities^7^ reflected increased rational use of medicines with laboratory use. All injections (four) cancelled after laboratory testing were for antibiotic treatment of STI in adults. Laboratory diagnosis may reduce drug costs if return visits for second or third treatments are avoided.

The presence of the study physician may have impacted on clinician practice in this study (Hawthorne effect).^14^ However, data collected during the overall 2-year study period showed clinical officers retained the level of patient referral to the laboratory in two health centres^5^ in the absence of the study physician. The proportion of patients referred for laboratory testing may therefore be used as an indicator of effective laboratory use.^5^ Although these data were collected some years ago, the findings are particularly relevant given the increasing recognition of the importance of more accurate patient diagnosis, rational drug use, quality health services and cost-effectiveness; and attempts by governments to develop effective diagnostic services at peripheral health care levels. Further studies are required to determine clinicians’ use of laboratory services in different health care settings and how diagnostic services can be designed for maximal utility and effectiveness.
